# SAMS-1 is required for the normal defecation motor program in *Caenorhabditis elegans*

**DOI:** 10.17912/micropub.biology.001101

**Published:** 2024-03-19

**Authors:** Keiko Hirota, Masato Matsuoka

**Affiliations:** 1 Department of Hygiene and Public Health, School of medicine, Tokyo Women's Medical University, Tokyo, Japan

## Abstract

Defecation is an ultradian rhythmic behavior in *Caenorhabditis elegans* . We investigated the involvement of *sams* family genes in regulating the defecation motor program. We found that *sams-1* mutants exhibited longer cycles than wild-type animals. With aging, the *sams-1* mutants also frequently skipped the expulsion (Exp) step of defecation behavior. The *sams-1* knockdown is known to reduce phosphatidylcholine (PC) levels, which are reversed by choline supplementation. We examined the effect of choline supplementation on defecation cycle times and Exp steps from adult days 1–4. Although choline supplementation did not alter the longer defecation cycle times of *sams-1* mutants, it restored the loss of the Exp step in *sams-1* mutants on adult days 3 and 4, suggesting a link between the regulation of the Exp step in *sams-1* mutants and PC production.

**Figure 1. Loss of function of sams-1 affects the defecation cycle time and Exp/pBoc ratio:**
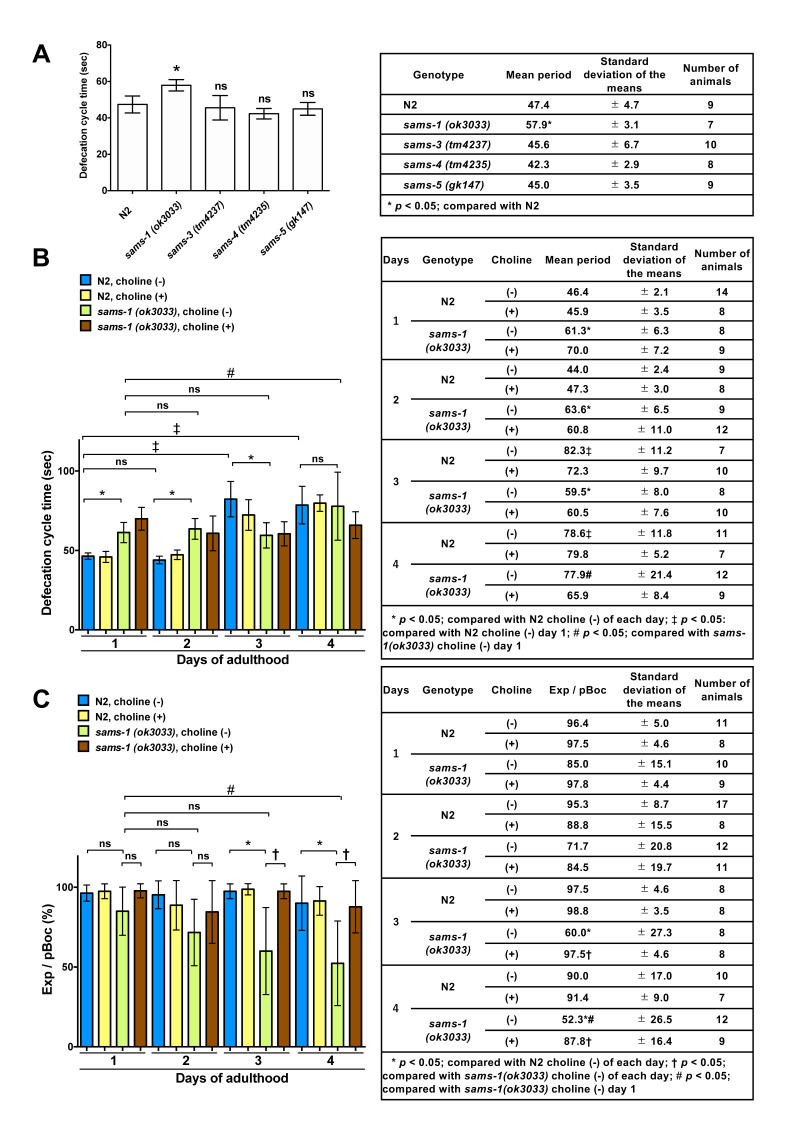
**(A)** Average defecation cycle times (mean ± standard deviation (SD)) for wild-type and mutant worms carrying *sams* family genes. The average defecation cycle time was determined by the time between pBoc steps. ns; the value is not significant. **(B)** Average defecation cycle times (mean ± SD) for wild-type and *sams-1* mutant worms in the absence or presence of 30 mM choline. The average defecation cycle time was defined as the time between pBoc steps. The time to reach adulthood was defined as “0 h.” Blue bars, representing defecation cycle time in N2 choline (-); yellow bars, representing N2 choline (+); green bars, representing *sams-1 ( ok3033 )* choline (-); and brown bars, representing *sams-1 ( ok3033 )* choline (+). ns; the value is not significant. **(C)** The percentage of defecation behaviors with Exp steps (mean ± SD) for wild-type and *sams-1* mutant worms in the absence or presence of 30 mM choline. The time to reach adulthood was defined as “0 h.” Blue bars, representing defecation cycle time in N2 choline (-); yellow bars, representing N2 choline (+); green bars, representing *sams-1 ( ok3033 )* choline (-); and brown bars, representing *sams-1 ( ok3033 )* choline (+). ns; the value is not significant.

## Description

Most animals exhibit rhythmic behaviors. In *Caenorhabditis elegans,* the defecation motor program (DMP) is a rhythmic behavior that occurs approximately every 45 s in well-fed young adult hermaphrodites. It involves three distinct contractions: a posterior body muscle contraction (pBoc), an anterior body muscle contraction (aBoc), and an enteric muscle contraction, which lead to the expulsion (Exp) of gut contents. DMPs are modulated by various environmental factors, such as temperature, food, and mechanical stimulation (Branicky and Hekimi, 2006; Liu and Thomas, 1994; Thomas, 1990) . Several metabolic pathways also affect defecation cycle times (Austin et al., 2010; Felkai, 1999; Kniazeva et al., 2003; Liu et al., 2012; Wong et al., 1995) . However, whether other metabolic pathways are involved in DMP regulation remains unclear.

*S* -adenosylmethionine (SAM) is synthesized from methionine, an essential amino acid, and adenosine triphosphate (ATP); the reaction is catalyzed by methionine adenosyl transferase (MAT) in mammals and SAM synthetase (SAMS) in *C. elegans* . SAM acts as a biological methyl donor, and the methyl portion of SAM is transferred to various substrates, such as proteins, DNA, and RNA. SAM and methionine compose the methionine cycle, contributing to multiple biosynthetic pathways, such as polyamine metabolism and transsulfuration (Parkhitko et al., 2019) . Among the four *sams* family genes, SAMS-1 is a major SAM synthetase (Cabreiro et al., 2013; Walker et al., 2011) . The *sams-1* loss-of-function or knockdown causes severe phenotypes, such as low phosphatidylcholine (PC) levels, increased lifespan, accumulation of lipid droplets, small body size, and lower number of eggs (Cabreiro et al., 2013; Ehmke et al., 2014; Hansen et al., 2005; Tamiya et al., 2013; Walker et al., 2011)

In the present study, we aimed to understand whether *sams* family genes are involved in regulating the DMP. We analyzed the defecation cycle time of adult day 2 of wild-type and four *sams* mutants: *sams-1 ( ok3033 )* , *sams-3 ( tm4237 )* , *sams-4 ( tm4235 ),* and *sams-5 ( gk147 )* . We found that only *sams-1 ( ok3033 )* exhibited significantly longer defecation cycle time than the wild-type ( Figure 1A ； N2 vs *sams-1 ( ok3033 )* , *p* < 0.05).

We analyzed the defecation cycle time from adult days 1 to 4. On adult days 1 and 2, *sams-1 ( ok3033 )* mutant exhibited longer cycles than wild-type animals [ Figure 1B ; N2 choline (-) day 1 vs *sams-1 ( ok3033 )* choline (-) day 1, *p* < 0.05; N2 choline (-) day 2 vs *sams-1 ( ok3033 )* choline (-) day 2, *p* < 0.05]. In wild-type, the defecation cycle times lengthened depending on the number of survival days, which is consistent with previous findings [ Figure 1B ; N2 choline (-) day 1 vs day 2, *not significant (ns)* ; day 1 vs day 3, *p* < 0.05; day 1 vs day 4, *p* < 0.05] (Bolanowski et al., 1981; Croll et al., 1977) . The defecation cycle time of *sams-1 ( ok3033 )* did not change until after adult day 3. On adult day 4, it was longer than that on adult day 1 [ Figure 1B ; *sams-1 ( ok3033 )* choline (-) day 1 vs day 4, *p* < 0.05]. The delay in the aging-dependent cycle time change of *sams-1 ( ok3033 )* may result from the longevity phenotype of *sams-1 ( ok3033 )* (Hansen et al., 2005) . The differences between the wild-type and the *sams-1 ( ok3033 )* on adult day 4 were not statistically significant [ Figure 1B ; N2 choline (-) day 4 vs *sams-1 ( ok3033 )* choline (-) day 4, *ns* ]. However, the Exp step was more frequently lost with aging in the *sams-1* mutants [ Figure 1C ; N2 choline (-) day 3 vs *sams-1 ( ok3033 )* choline (-) day 3, *p* < 0.05; N2 choline (-) day 4 vs *sams-1 ( ok3033 )* choline (-) day 4, *p* < 0.05].

The knockdown of *sams-1* reduces SAM levels and SAM-dependent methylation required for PC synthesis (Li et al., 2011; Walker et al., 2011) . Choline supplementation forces the Kennedy pathway to produce PC, restoring the PC synthesis-dependent phenotype (Ding et al., 2015; Li et al., 2011; Palavalli et al., 2006; Walker et al., 2011) . To determine whether longer defecation cycle times and the loss of the Exp step in *sams-1 ( ok3033 )* were PC-dependent, we examined the effects of choline supplementation on DMP of *sams-1 ( ok3033 )* from adult days 1 to 4. Choline supplementation did not affect the defecation cycle time of the wild-type animals and *sams-1 ( ok3033 )* on either day [ Figure 1B ; day 1 - day 4, N2 choline (-) vs. N2 choline (+), *ns* ; *sams-1 ( ok3033 )* choline (-) vs. *sams-1 ( ok3033 )* choline (+), *ns* ]. However, the Exp/pBoc ratio of *sams-1 ( ok3033 )* on adult days 3 and 4 was rescued under choline supplementation [ Figure 1C ; *sams-1 ( ok3033 )* choline (-) day 3 vs *sams-1 ( ok3033 )* choline (+) day 3, *p* < 0.05; *sams-1 ( ok3033 )* choline (-) day 4 vs *sams-1 ( ok3033 )* choline (+) day 4, *p* < 0.05]. Therefore, these data indicate that the mechanisms whereby the loss of function of *sams-1* decreases the Exp/pBoc ratio on adult days 3 and 4 are linked to PC levels. These findings contribute to our understanding of the complex interplay between the PC synthesis pathway and the regulation of DMP in *C. elegans* .

## Methods

***C. elegans* strains and culture**

The strains were maintained as described previously (Hirota and Matsuoka, 2021) . The Bristol N2 strain was used as the wild-type strain in this study. Bristol N2 was obtained from the *Caenorhabditis* Genetics Center. The bacterial strain used as a food source for *C. elegans* was *E. coli* OP50 . *sams-1 ( ok3033 )* was outcrossed five times, and *sams-3 ( tm4237 ), sams-4 ( tm4235 )* , and *sams-5 ( gk147 )* were outcrossed three times with the N2 . The outcrossed mutants were kindly provided by Professor Akiyoshi Fukamizu (University of Tsukuba).

**Analysis of the defecation motor program**

For the defecation cycle time and the Exp/pBoc ratio analysis, wild-type N2 animals or *sams* mutants maintained on nematode growth media (NGM) petri plates seeded with *E. coli* strain OP50 at 20°C were used. The defecation cycle times and the Exp/pBoc ratios of wild-type and mutant strains on NGM plates seeded with *E. coli* strain OP50 were scored at 22°C–25°C. For each strain, 7–17 worms were analyzed. One animal was placed on a 9 cm nematode growth medium (NGM) plate. After an acclimation period of at least 10 min, the time interval between pBoc steps was calculated as the average time per 11 consecutive pBoc step. The pBoc steps were distinct and could be reliably assessed. For individuals that defecated less than 10 times in 20 min, the average time of defecation during that time was calculated and plotted. The Exp/pBoc ratio was calculated from 10 defecation cycle periods. The defecation cycle scoring system developed by Dr. Motomichi Doi (National Institute of Advanced Industrial Science and Technology) was kindly provided and used to record the pBoc and expulsion times.

**Choline supplementation**

Choline chloride (product No. 67-48-1; Sigma-Aldrich, St. Louis, MO, USA) was added to the NGM before pouring onto the plates. Synchronized L1 larvae were incubated on NGM plates corresponding to different experimental groups (i.e., control and 30 mM choline chloride plates) until adulthood. Each day, one nematode was transferred to the NGM plate in the presence or absence of 30 mM choline chloride for the assay, and the defecation cycle time was counted.

**Statistical analysis**

Results are presented as the mean ± standard deviation (SD). The statistical significance of the data presented in Figure 1 was determined using one -way analysis of variance, followed by Tukey's test using GraphPad Prism software ver. 6. A *p* value < 0.05 was considered significant.

## Reagents

**Table:** 

Strain	Genotype	Available from
N2	wild type	CGC
TKB458	*sams-1 ( ok3033 ) X *	This study
TKB148	*sams-3 ( tm4237 ) IV *	Tamiya et al. 2013
TKB150	*sams-4 ( tm4235 ) IV *	Tamiya et al. 2013
TKB151	*sams-5 ( gk147 ) IV *	Tamiya et al. 2013
